# Tin-Doped LATP Electrolytes Incorporated with Nickel-Rich Ternary Cathodes for Solid-State Pouch Cells Exhibiting High-Rate Capability and Excellent Cycling Stability

**DOI:** 10.1021/acsomega.6c04655

**Published:** 2026-07-17

**Authors:** Pradeep Kumar Panda, Yun-Ruei Huang, Siyong Gu, Pranjyan Dash, Congrui Jin, Jianlin Li, Chien-Te Hsieh

**Affiliations:** † Department of Chemical Engineering and Materials Science, 34895 Yuan Ze University, Taoyuan 32003, Taiwan; ‡ Key Laboratory of Functional Materials and Applications of Fujian Province, School of Materials Science and Engineering, Xiamen University of Technology, Xiamen 361024, China; § Department of Chemical Engineering and Biotechnology, National Taipei University of Technology (Taipei Tech), Taipei 10608, Taiwan; ∥ Department of Engineering Technology and Industrial Distribution, 14736 Texas A&M University, College Station, Texas 77843, United States; ⊥ Applied Materials Division, 1291 Argonne National Laboratory, Lemont, Illinois 60439, United States; # Department of Mechanical, Aerospace, and Biomedical Engineering, University of Tennessee, Knoxville, Tennessee 37996, United States

## Abstract

This study explores the use of Sn-doped sodium superionic conductor (NASICON)-type Li_1+*x*_Al_*x*_Ti_2–*x*_(PO_4_)_3_ (LATP) electrolytes in solid-state pouch cells with Ni-rich ternary cathodes that exhibit high-rate capability, low inner resistance, and superior cyclability. The pouch cell configuration includes two types of Ni-rich NCM cathodes, LiNi_0.5_Co_0.2_Mn_0.3_O_2_ (NCM523) and LiNi_0.6_Co_0.2_Mn_0.2_O_2_ (NCM622), paired with artificial graphite anodes (MG11) and synthesized LATP-containing composite solid electrolytes (CSEs). These cells demonstrate superior cyclic performance with high energy and power densities. Sol–gel and thermal calcination produced highly ionic conductivity Sn-LATP powders for CSEs. The equivalent ionic radii of Sn^4+^ and Ti^4+^ in NASICON-type lattices reduce distortion, but Sn^4+^’s greater Pauling electronegativity (∼1.96) improves the stability. The Sn-LATP powders in CSEs enhance the high-rate capabilities and cyclic stability of the solid-state pouch cells. NCM523 and NCM622 pouch cells with cathode-equipped pouches showed enhanced rate capability, with NCM523 achieving 58.9% capacity retention at 3C to 0.2C and NCM622 achieving 91.1% retention and ∼99.5% Coulombic efficiency over 200 cycles. Sn-LATP powders dramatically lower ESR values, according to the equivalent circuit impedance analysis. The diffusion coefficients for NCM523||Sn-LATP||MG11 and NCM622||Sn-LATP||MG11 pouch cells are 2.18 × 10^–8^ cm^2^ s^–1^ and 1.47 × 10^–8^ cm^2^ s^–1^, respectively, while the energy densities of NCM523||Sn-LATP||MG11 and NCM622||Sn-LATP||MG11 pouch cells are 132 and 172 Wh kg^–1^ at high-power densities of 717 and 778 W kg^–1^, respectively. The optimum CSE layer design using Sn-LATP particles presented in this work may lead to enhanced solid-state energy storage devices.

## Graphical abstract

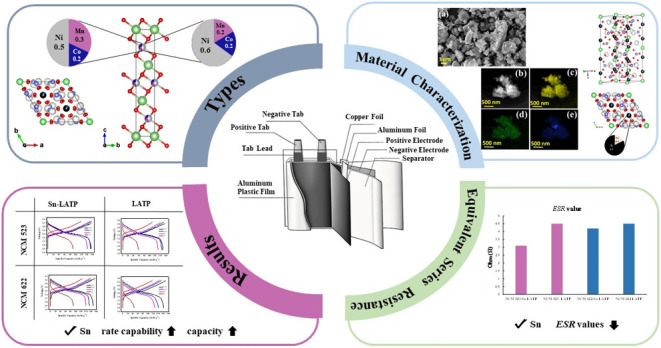

## 1 Introduction

Lithium-ion batteries (LIBs) are now necessary in contemporary life, powering consumer devices, electric automobiles, and aerospace goods.^1^ As the demand for electrification increases, LIB adoption is surging. Technological advancements have gradually increased the energy density of commercial LIBs to 220–300 Wh kg^–1^, with state-of-the-art versions targeting 350–500 Wh kg^–1^.^2^ This rising demand underscores the need for LIBs that are not only safe but also offer high energy densities. However, current LIBs with liquid electrolytes pose substantial risks due to flammable organic solvents, which can lead to fires or even explosions. The development of solid-state electrolytes (SSEs) to replace liquid electrolytes is a viable technique for improving the safety of LIBs by reducing flammability and electrolyte leakage. As a result, all solid-state batteries are regarded as crucial future technologies and have garnered significant interest in recent years.^3–5^

To address LIB safety concerns, the focus has shifted to developing SSEs,^6,7^ which can broadly be divided into two types: inorganic ceramics and solid polymer electrolytes.^8–10^ Inorganic SSEs are particularly promising due to their high thermal stability, nonflammability, and the absence of leakage and volatilization risks.^11,12^ Many inorganic SSEs, including garnets, sodium superionic conductors (NASICON), amorphous oxides, and perovskites, exhibit excellent thermal stability and room-temperature ionic conductivities compared to typical liquid electrolytes.^13^ Among these, NASICON-type SSEs, specifically Li_1+*x*_Al_*x*_Ti_2–*x*_(PO_4_)_3_ (LATP) with *x* = 0.3, demonstrate high ionic conductivity (10^–4^ to 10^–3^ S cm^–1^),^14,15^ robust mechanical properties, and a wide electrochemical window.^16–18^ Recently, Salazar et al. investigated CSEs combined with Li_1.5_Al_0.5_Ti_1.5_(PO_4_)_3_ ceramics using the cold sintering process with other raw materials. However, the ionic conductivities range between 10^–4^ and 10^–3^ S cm^–1^ at room temperature, and the discharge capacity was 141 mAh. g^–1^ at C/10 rate after 50 cycles.^19^ Similarly, Liu et al. studied the interfacial stability between NASICON-type solid-state electrolytes and lithium metal anodes. However, this study used a composite electrolyte for the Li_4_Ti_5_O_12_ (LTO) interlayer for the evaluation of the device performance.^20^

A previous study effectively proved that doping is a feasible method for the enhancement of ionic conductivity of LATP for the electrodes, which not only improves the ionic conductivity but also enhances the excellent cyclic performance in energy storage devices.^21,22^ Among all the doped metals, tin (Sn) exhibits superior performance in terms of ionic conductivity and overall battery performance. Sn-based solid-state batteries attain an ionic conductivity of 1.88 × 10^−4^ S cm^–1^ at room temperature. High ionic conductivity was achieved owing to the alteration of the lattice parameters and fast lithium-ion transportation. Overall battery performance, such as capacity retention, was achieved at 86% at 0.2C and high columbic efficiency (>99.0%) over 500 cycles.^21^ Xu et al. synthesized various contents of Sn-doped CSEs for the enhancement of LiFePO_4_-based battery. However, the capacity retention at low and high rates and ionic conductivity were not satisfactory.^23^ Similarly, Jiang et al. studied the electrochemical properties of Sn-doped NaTi_2_(PO_4_)_3_/C and found that doping resulted in outstanding electrochemical performance and suitable anode candidates for Li-ion batteries.^24^ Based on these findings, Sn-doped LATP powder is a viable component of composite solid-state electrolytes (CSEs) for high-performance solid-state batteries. However, there are few reports on the application of Sn-doped LATP powders in solid-state pouch cells using commercially available ternary cathode materials such as LiNi_*x*_Co_*y*_Mn_1–*x*–*y*_O_2_ (NCM > 50% Ni). To advance practical applications, this study aims to develop solid-state pouch cells using two Ni-rich NCM cathodes, LiNi_0.5_Co_0.2_Mn_0.3_O_2_ (NCM523) and LiNi_0.6_Co_0.2_Mn_0.2_O_2_ (NCM622), paired with artificial graphite anodes and LATP-containing CSEs, offering excellent cyclic performance with high energy and power densities. A comparative study was conducted to investigate the battery performance of pouch cells with NCM523 and NCM622 cathodes. We engineered an optimal CSE configuration, comprising metal-doped LATP powders, PVDF-HFP, and Li salt, for high-performance solid-state pouch cells. A robust battery structure with minimal internal resistance and exceptional capacity retention over continuous cycling is confirmed by the CSEs’ efficiency in preventing Li dendrite formation and their excellent compatibility with pouch cells.

## 2 Experimental Section

The synthesis of Sn-doped LATP powders, assembly of solid-state pouch cells, material characterization, and electrochemical analysis are provided in the Supporting Information.

## 3 Results and Discussion

### 3.1 Material Characterization

The XRD patterns of both pristine LATP powders and LATP powders doped, derived from the sol–gel synthesis method, are presented in Figure 1. The LATP powders display a highly crystalline structure with the XRD pattern aligning well with the standard NASICON-type structure (rhombohedral lattice, JCPDS PDF # ICDD 00-035-0754).^25–27^ All the characteristic peaks at 2-theta are positioned at 20.8, 24.5, 29.7, and 33.3°, which correspond to the crystalline planes of (104), (113), (024), and (116), respectively.^28,29^ The XRD pattern of the Sn-LATP powders also displays all the diffraction peaks associated with the rhombohedra *R3c* NASICON-type structure, indicating a pure crystalline phase without impurity peaks such as AlPO_4_.^30^ To investigate the impact of Sn dopants on the crystalline structure, the crystalline sizes and lattice constants of the pristine and Sn-LATP powders are tabulated in Table 1. The mean crystalline size (*D*_xrd_) of the Sn-LATP powders was estimated using Scherrer’s formula *D*_xrd_ = 0.9 λ/β_1/2_ cos θ, where *D*_xrd_ represents the average crystalline size (nm), λ signifies the wavelength of X-ray (0.15406 nm), θ indicates the angle at the peak maximum, and β_1/2_ describes the full width at half-maximum (FWHM).^31^ The FWHM of the Sn-LATP powders is significantly wider than that of pristine LATP, indicating reduced crystallinity due to Sn doping. The *D*_xrd_ values decrease from 81.3 nm (pristine LATP) to 45.2 nm (Sn-LATP), attributed to the incorporation of Sn dopants, which likely causes lattice distortions and increased grain boundaries. Table 1 shows a slight reduction in the lattice constant (*L_c_*) from 25.83 Å (pristine LATP) to 25.77 Å (Sn-LATP), with a 0.35% deviation in the other lattice constant (*L*_a_) for comparison between the pristine LATP and Sn-LATP powders. The substitution of Ti^4+^ ions with Sn^2+^ ions in LiTi_2_(PO_4_)_3_ aligns with previous findings,^1,10^ with the Ti–Sn bond length estimated at 2.73 Å compared to the Ti–Ti bond length of 3.07 Å, as programmed by The Materials Project.^32–34^ These findings indicate the potential changes in lattice parameters due to dopants. However, the similar ionic radii of Sn^4+^ and Ti^4+^ prevent excessive lattice distortion, while the higher Pauling electronegativity of Sn^4+^ (∼1.96) enhances the NASICON framework’s stability.^22^

**Figure 1:**
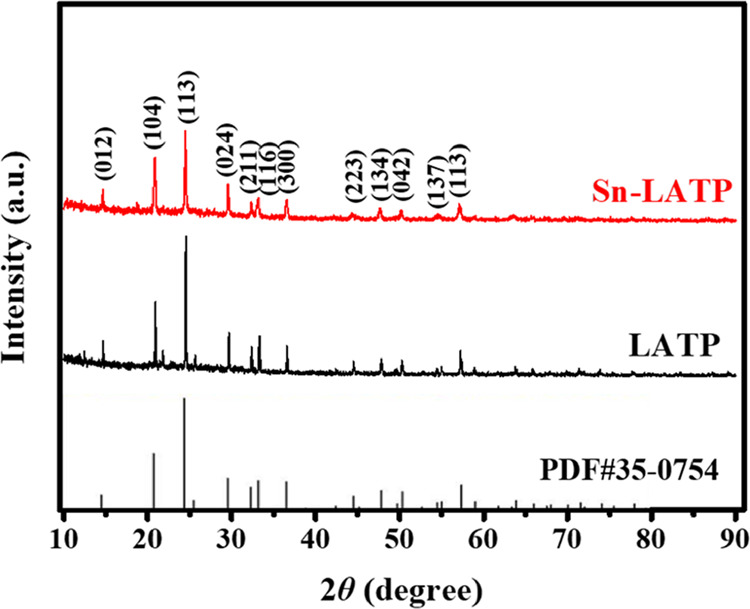
Typical XRD patterns of Sn-LATP and LATP powders with standard patterns.

**Table 1:** Lattice Parameters, Crystalline Sizes, and *d*-Spacing Distances of LATP and Sn-LATP Powders

				lattice parameters (Å)		
sample	2-theta (deg.)	D_113_ (nm)	*D*_xrd_ (nm)	a = b	c	deviation (%)
LATP	24.52	0.363	81.30	5.66	25.83	
Sn-LATP	24.56	0.363	45.16	5.68	25.77	0.35

The morphology of the sample is examined using SEM and TEM analyses. Figure 2a shows the SEM image of the Sn-doped LATP powder samples, which exhibited that nanoparticles are clustered with a particle size of 1.2 to 2.5 μm. The TEM micrograph (Figure 2b) confirms that the LATP cluster consists of rhombohedral-shaped crystals. Further, energy-dispersive X-ray spectroscopy (EDS) was performed to evaluate the presence of elements with the atomic ratios. Figure 2c–e displays the EDS mapping of the Sn-doped LATP sample, which highlights the presence of dopants (yellow, green, and blue dots) in the LATP powders. The above results prove that O, Sn, and Ti elements are well dispersed within the Sn-LATP crystals. The EDS analysis also provided the atomic ratio (dopant concentration), as shown in Table 2, confirming successful Sn doping into the LATP lattices, with a Sn dopant concentration of approximately 7.16 wt % and a Sn/Ti atomic ratio of 57.4 atomic % in the Sn-LATP powders. Further, surface and cross-sectional SEM images of Sn-LATP slurry coated on NCM622 are shown in Figure S1to elucidate the morphology of the materials.

**Figure 2:**
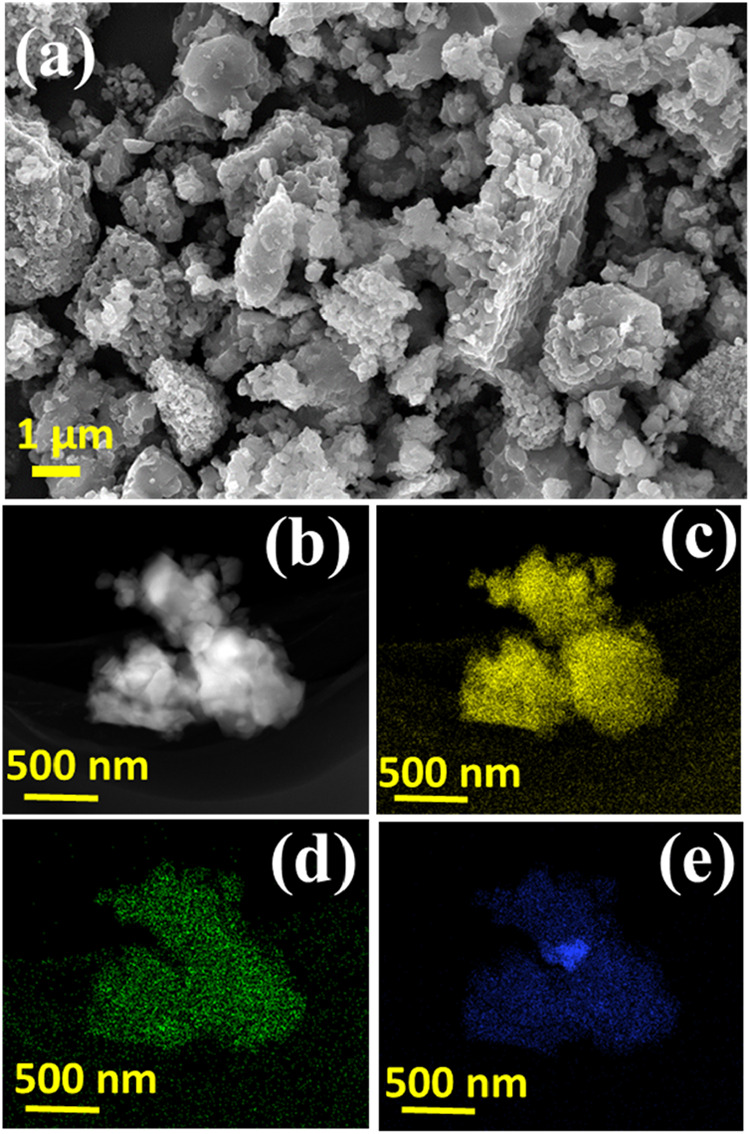
(a) SEM image and (b) TEM micrograph of Sn-LATP powders, and elemental mapping images of Sn-LATP powders: (c) O, (d) Sn, and (e) Ti.

**Table 2:** Chemical Composition (Weight % and Atomic %) of Sn-LATP Powders

elements	weight (%)	atomic (%)
Ti	14.06	9.4
O	46.76	58.7
Al	6.16	7.2
P	25.86	19.3
Sn	7.16	5.4

### 3.2 Electrochemical Performance of Pouch Cells Using NCM523 Cathodes

Figure 3a,b illustrates the typical charge–discharge curves of pouch cells (i.e., NCM523||Sn-LATP||MG11 and NCM523||LATP||MG11) at various C rates (0.1 to 3C). Both pouch cells display symmetric charge–discharge profiles within a potential window of 2.8–4.3 V. The NCM523||Sn-LATP||MG11 pouch cell displayed an initial discharge capacity of approximately 158 mAh g^–1^ at 0.1C, slightly higher than that of the NCM523||LATP||MG11 cell (∼150 mAh g^–1^ at 0.1C). At 3C, the discharge capacities decrease by approximately 90.5 and 60.4 mAh g^–1^ for the NCM523||Sn-LATP||MG11 and NCM523||LATP||MG11 pouch cells, respectively. The improved rate capability of the Sn-LATP powders suggests their feasibility for high-power energy device applications. The fast reversible capacity is attributed to the redox couple of Ni^2+/4+^, while Mn^4+^ remains electrochemically inactive. The Co^3+/4+^ couple is absent since its redox reaction takes place at potentials above 4.6 V.^35,36^ Thus, only the Ni^2+/4+^ redox reaction contributes to the symmetric charge–discharge profile with a high reversible capacity for both pouch cells, indicating excellent lithiation/delithiation process reversibility in CSEs. The thermodynamic formulation of the Li insertion/extraction process in the NCM523 cathode is as follows^37–39^

R1 $${\mathrm{LiNi}}_{0.5}{\mathrm{Co}}_{0.2}{\mathrm{Mn}}_{0.3}O_{2}↔{\mathrm{Li}}_{1-x}{\mathrm{Ni}}_{0.5}{\mathrm{Co}}_{0.2}{\mathrm{Mn}}_{0.3}O_{2}+{xLi}^{+}+{xe}^{-}$$

**Figure 3:**
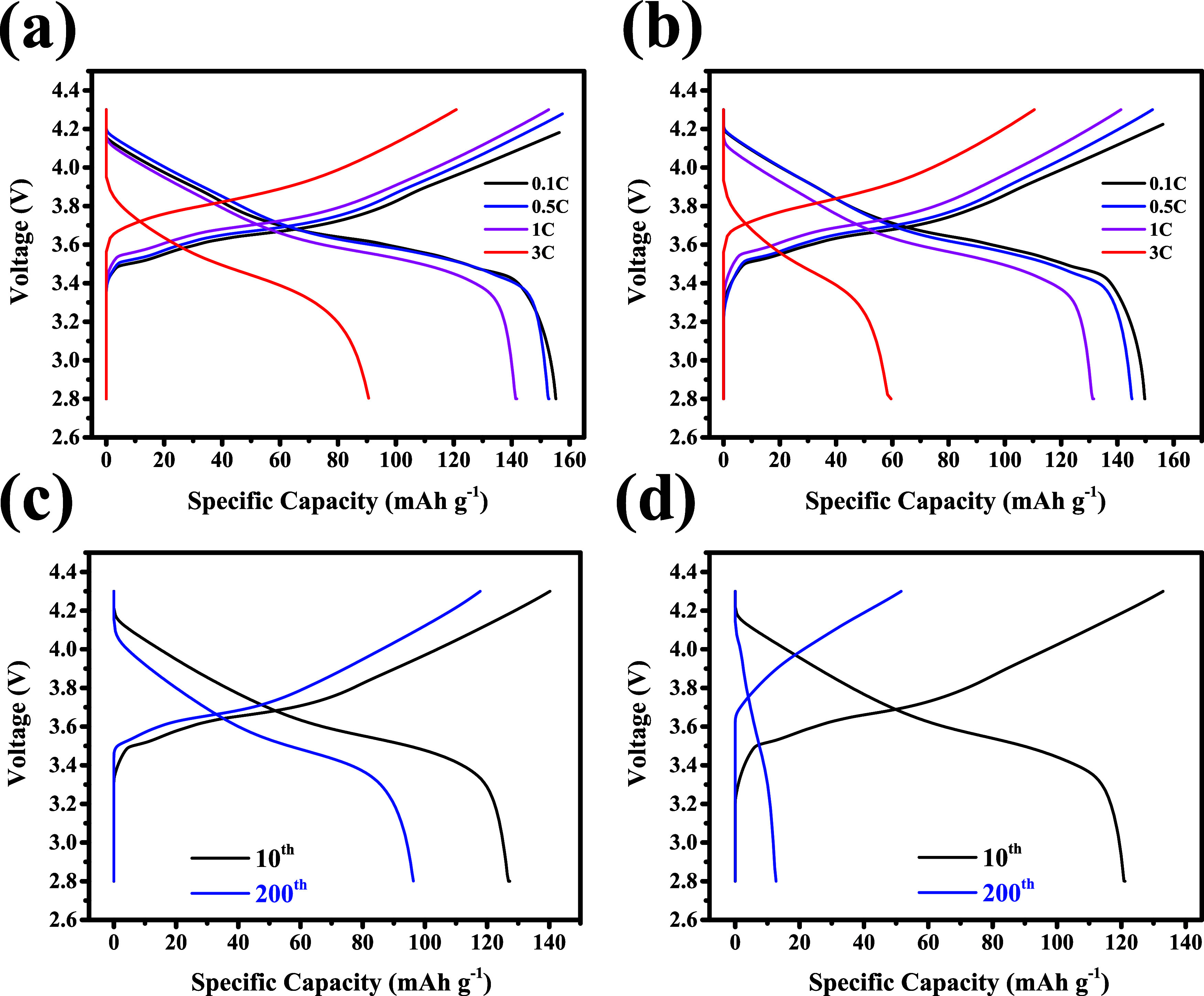
Typical charge/discharge curves at various C rates: (a) NCM523||Sn-LATP||MG11 and (b) NCM523||LATP||MG11. The charge/discharge curves of pouch cells at 0.2C (charging)/1C (discharging): (c) NCM523||Sn-LATP||MG11 and (d) NCM523||LATP||MG11.

The *x* value is contingent on the charged and discharged states of the NCM cathodes across various potential ranges. The presence of Sn-LATP powders in the SSEs positively enhances the reversible capacity and high-rate capability. The improved performance suggests that Sn dopants facilitate an enhanced *x* value, especially at high rates, leading to faster chemical reaction kinetics and lower diffusion resistance due to the higher ionic conductivity in CSEs. Figure 3c,d shows the typical charge–discharge curves of both solid-state pouch cells at 0.2C (charging)/1C (discharging). With increasing cycle number, the NCM523||Sn-LATP||MG11 pouch cell still maintains superior capacity retention after 200 cycles compared to the NCM523||LATP||MG11 cell. After 200 cycles, the NCM523||Sn-LATP||MG11 pouch cells achieve a capacity retention of 82.2% with a high Coulombic efficiency (∼99.5%), as shown in Figure 4a,b. In contrast, the NCM523||LATP||MG11 cell demonstrates inferior cyclic performance, including reduced capacity retention and Coulombic efficiency after 200 cycles. This performance deterioration suggests that the unmodified LATP CSE suffers significantly from lithium dendrite formation after 200 cycles, resulting in inadequate cyclic stability and inferior rate capability. However, it is notable that the capacity retention and Coulombic efficiency of solid-state pouch cells were drastically improved with Sn-LATP powders. The enhanced cyclic performance originates mainly from the increased ionic conductivity and reduced resistance, which mitigates polarization within the CSE and inhibits the growth of Li-dendrites during the charge–discharge cycling process. Moreover, the presence of Sn^4+^, characterized by a higher Pauling electronegativity of approximately 1.96, enhances the stability of the NASICON framework, resulting in remarkable cyclic performance.^40,41^ Figure 4c,d highlights the relationship between the discharge capacity and C rate for the two solid-state pouch cells. The NCM523||Sn-LATP||MG11 pouch cell displays a superior rate capability as compared to the NCM523||LATP||MG11 cell. Especially, the rate capability of solid-state pouch cells was significantly enhanced at high rates, i.e., the capacity ratio at 3C to 0.2C is 58.9% (NCM523||Sn-LATP||MG11) > 39.8% (NCM523||LATP||MG11). The CSE containing the Sn-LATP particles demonstrated high ionic conductivity (∼1.88 × 10^–4^ S cm^–1^) at ambient temperature.^21^ The improved ionic conductivity is due to the Sn dopant, which distorts the lattice, modifies the Li-ion transport channels, and increases the Li^+^ concentration. Based on the experimental results, the Sn-LATP conductor is a promising candidate for NASICON-type conductors for SSEs to promote the reversible capacity, rate capability, and cyclic stability of solid-state batteries.

**Figure 4:**
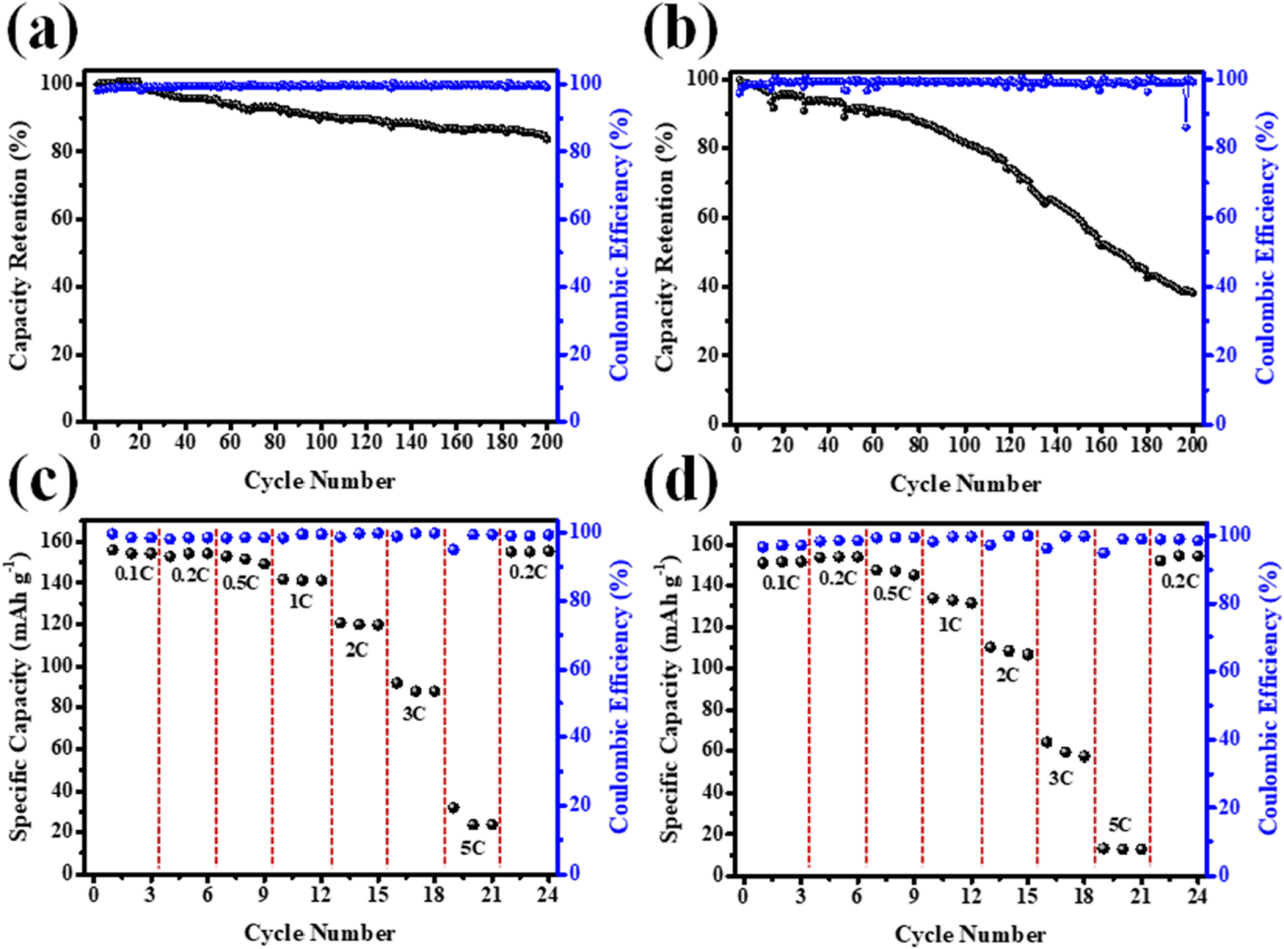
Cyclic performance of pouch cells at 0.2C (charging) and 1C (discharging): (a) NCM523||Sn-LATP||MG11 and (b) NCM523||LATP||MG11; and variation in discharge capacity with various C rates of pouch cells: (c) NCM523||Sn-LATP||MG11 and (d) NCM523||LATP||MG11.

### 3.3 Electrochemical Performance of Pouch Cells Using NCM622 Cathodes

Figure 5a,b presents the typical charge–discharge curves of pouch cells (NCM622||Sn-LATP||MG11 and NCM622||LATP||MG11) at various rates from 0.1 to 3C. The pouch cells were charged and discharged at voltages of 2.8 to 4.3 V. Both the NCM622||Sn-LATP||MG11 and NCM622||LATP||MG11 pouch cells displayed high specific capacities of approximately 169 and 161 mAh g^–1^ at 0.1C, respectively. At 3C, the discharge capacities of the NCM622||Sn-LATP||MG11 and NCM622||LATP||MG11 pouch cells decreased to 92.8 and 45.4 mAh g^–1^, respectively. Again, the rate capability of the solid-state pouch cells is strongly enhanced by Sn doping in the LATP lattices. Figure 5c,d illustrates the typical charge–discharge curves of both solid-state pouch cells at 0.2C (charging)/1C (discharging), showing a symmetric charge–discharge profile.

**Figure 5:**
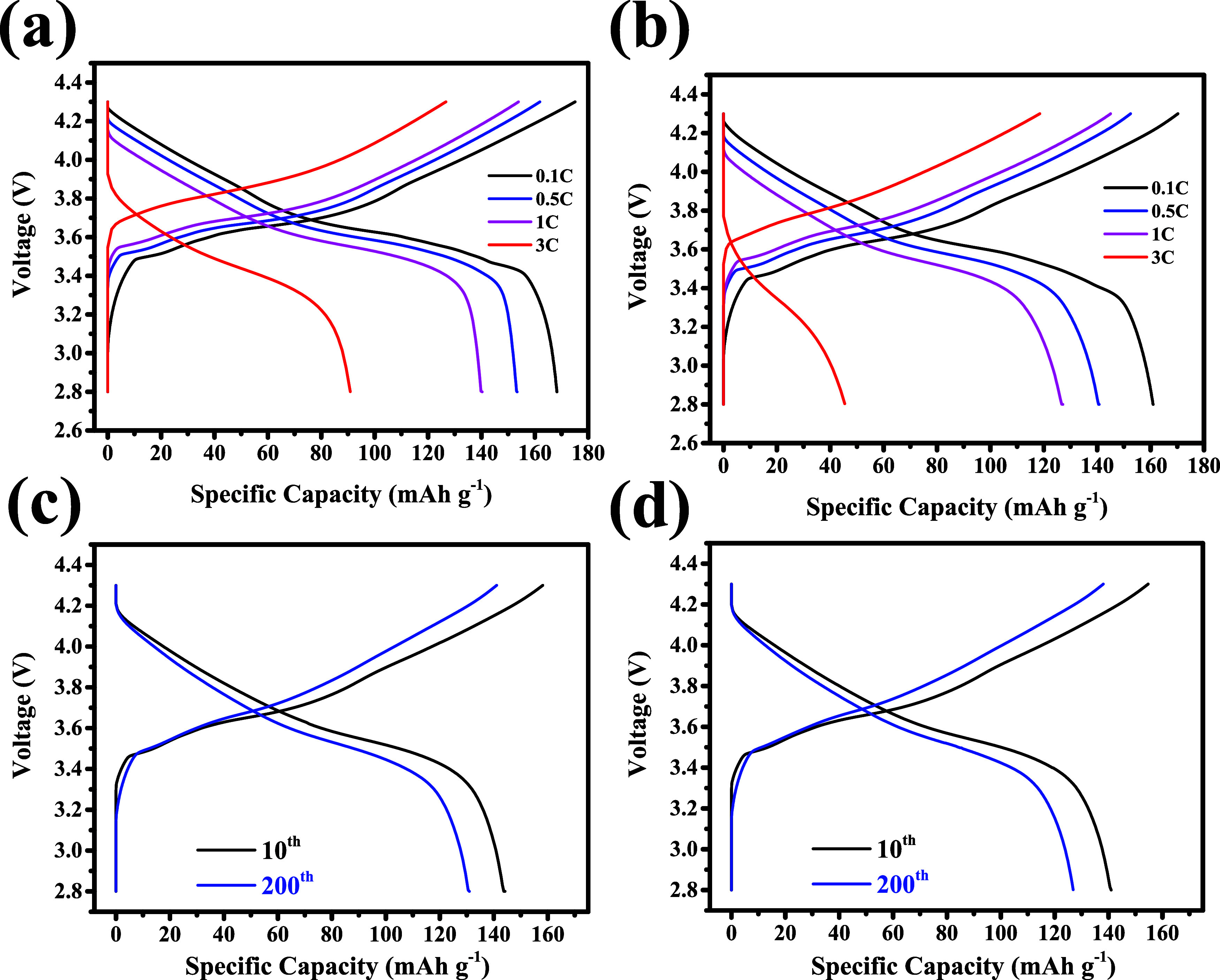
Typical charge/discharge curves at various C rates: (a) NCM622||Sn-LATP||MG11 and (b) NCM622||LATP||MG11. The charge/discharge curves of pouch cells: (c) NCM622||Sn-LATP||MG11 and (d) NCM622||LATP||MG11.

Figure 6a,b shows capacity retention and Coulombic efficiency as functions of the cycle number. Interestingly, both solid-state pouch cells exhibit excellent cyclic stability within 200 cycles. The capacity retention of the NCM622||Sn-LATP||MG11 pouch cell reached 91.1% with a high Coulombic efficiency (∼99.5%) after 200 cycles. Moreover, the NCM622||LATP||MG11 cell also displays excellent cyclic performance, including capacity retention (∼88.2%) and Coulombic efficiency (∼99.2%) after 200 cycles. As shown in Figure 6c,d, the variation in the discharge capacity with the C rate for both solid-state pouch cells confirms the superior rate capability of the NCM622||Sn-LATP||MG11 pouch cell, i.e., the capacity ratio at 3C to 0.2C: 54.8% (NCM622||Sn-LATP||MG11) > 21.5% (NCM622||LATP||MG11). Clearly, the CSEs containing Sn-LATP powders are suitable for both NCM523 and NCM622 cathodes. It is generally recognized that Li–Sn bonding formation with negative formation energy (−0.326 eV/atom) is a spontaneous process favorable for Li ionic conduction.^35^ Consequently, this optimistic result (i.e., improved cell performance at a high C rate) emphasizes that NCM622-supported CSEs facilitate the development of high-power solid-state batteries.

**Figure 6:**
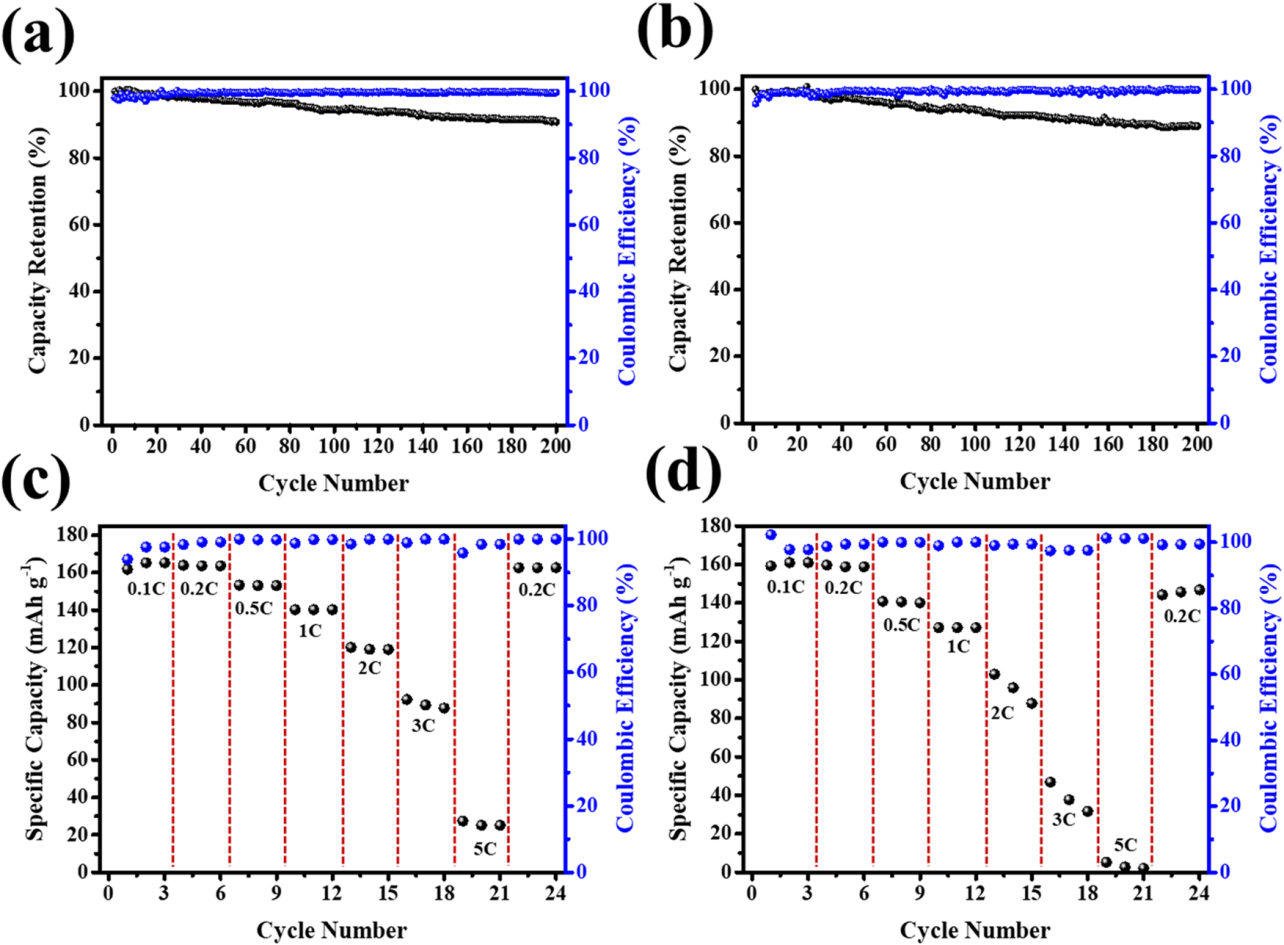
Cyclic performance of pouch cells at 0.2C (charging) and 1C (discharging): (a) NCM622||Sn-LATP||MG11 and (b) NCM622||LATP||MG11, and variation of discharge capacity with C rate of pouch cells: (c) NCM622||Sn-LATP||MG11 and (d) NCM622||LATP||MG11.

The different electrochemical behaviors of the NCM523 and NCM622 pouch cells can be mainly attributed to the variation in the Ni content. Increasing the Ni fraction from NCM523, LiNi_0.5_Co_0.2_Mn_0.3_O_2_, to NCM622, LiNi_0.6_Co_0.2_Mn_0.2_O_2_ increases the number of electrochemically active Ni redox centers. Within the operating voltage window of 2.8–4.3 V, the reversible capacity is primarily governed by the Ni/Ni^4+^ redox reaction, while Mn^4+^ remains largely electrochemically inactive and Co redox contributes less significantly under this voltage range. Therefore, the higher Ni content in NCM622 enables a higher discharge capacity and energy density compared to NCM523. This is evidenced by the higher initial discharge capacity of the NCM622||Sn-LATP||MG11 pouch cell, approximately 169 mAh g^–1^ at 0.1C, compared with approximately 158 mAh g^–1^ for the NCM523||Sn-LATP||MG11 cell. The same trend is observed in the Ragone analysis, where the NCM622||Sn-LATP||MG11 pouch cell delivers approximately 172 Wh kg g^–1^ at 778 W kg g^–1^, exceeding the 132 Wh kg^–1^ obtained for the NCM523||Sn-LATP||MG11 cell at 717 W kg^–1^. However, the increased Ni content also affects the reaction kinetics and the interfacial behavior. The NCM622||Sn-LATP||MG11 cell exhibits a slightly higher ESR value of 4.2 Ω and a lower Li-ion diffusion coefficient of 1.47 × 10^–8^ cm^2^ s^–1^ than those of the NCM523||Sn-LATP||MG11 cell, which has an ESR of 3.1 Ω and a diffusion coefficient of 2.18 × 10^–8^ cm^2^ s^–1^. These results suggest that although a higher Ni content improves the capacity and energy output, it may also increase the polarization and interfacial resistance during Li^+^ extraction/insertion. Nevertheless, the Sn-LATP-containing composite solid electrolyte effectively alleviates these kinetic limitations by reducing the internal resistance and improving the Li-ion transport. As a result, the NCM622||Sn-LATP||MG11 pouch cell maintains superior cycling stability, achieving 91.1% capacity retention with an approximate 99.5% Coulombic efficiency after 200 cycles. Therefore, the performance comparison demonstrates that NCM523 offers relatively lower resistance and faster Li-ion diffusion, whereas NCM622 provides higher capacity and energy density due to its larger Ni redox contribution. The Sn-LATP-based CSE plays a key role in balancing these effects and enabling stable high-rate operation in both Ni-rich cathode systems.

### 3.4 EIS Analysis and Ragone Plots of Solid-State Pouch Cells

The Nyquist plots of the NCM523||Sn-LATP||MG11 and NCM523||LATP||MG11 pouch cells at 3.6 V are shown in Figure 7a. The Nyquist plots illustrate three distinct regions: a single intersection along the real axis at a high frequency, two depressed semicircles at a medium frequency, and a slant line at a low frequency.^42,43^ The Nyquist plots are similar for both the NCM523 and NCM622 cathodes, as illustrated in Figure 7b. In general, a single depressed semicircle identified throughout the entire frequency range is attributed to the charge-transfer impedance at the interface between the electrode and electrolyte.^41,44^ To describe the impedance behavior, an equivalent circuit model (see the inset of Figure 7b) is proposed, consisting of the CSE bulk resistance (*R1*), interfacial solid-electrolyte interphase (SEI) resistance between CSEs and electrodes (*R2*) in parallel with first constant phase element (*CPE1*, for the capacitances of the SEI film), charge-transfer resistance (*R3*) in parallel with the second constant phase electrode (*CPE2*, for the capacitances of the double layer), and diffusional impedance by the Warburg element (*W*).^45^ The *Z*-view software package was utilized for EIS analysis of these solid-state pouch cells. The impedance spectra recorded and the model predictions for all pouch cells exhibited deviations of less than 10% throughout the entire frequency range. The overall internal resistance of the pouch cells was calculated by assessing the equivalent series resistance, *ESR* (= *R1* + *R2* + *R3*).^46,47^ The *ESR* values of NCM523||Sn-LATP||MG11, NCM523||LATP||MG11, NCM622||Sn-LATP||MG11, and NCM622||LATP||MG11 are determined to be 3.1, 4.5, 4.2, and 5.4 Ω, respectively. Variations in the *ESR* values among the pouch cells reveal that the Sn-LATP filler in the CSEs alleviates battery polarization, enabling fast ionic transport and charge transfer. The *ESR* value (i.e., the overall resistance) is significantly decreased by the robust design of CSEs incorporated with Sn-LATP powders as compared to that with pristine LATP, attributed to the substantial alleviation of the bulk electrolyte, charge transfer, and interfacial electrolyte/electrode impedance.

**Figure 7:**
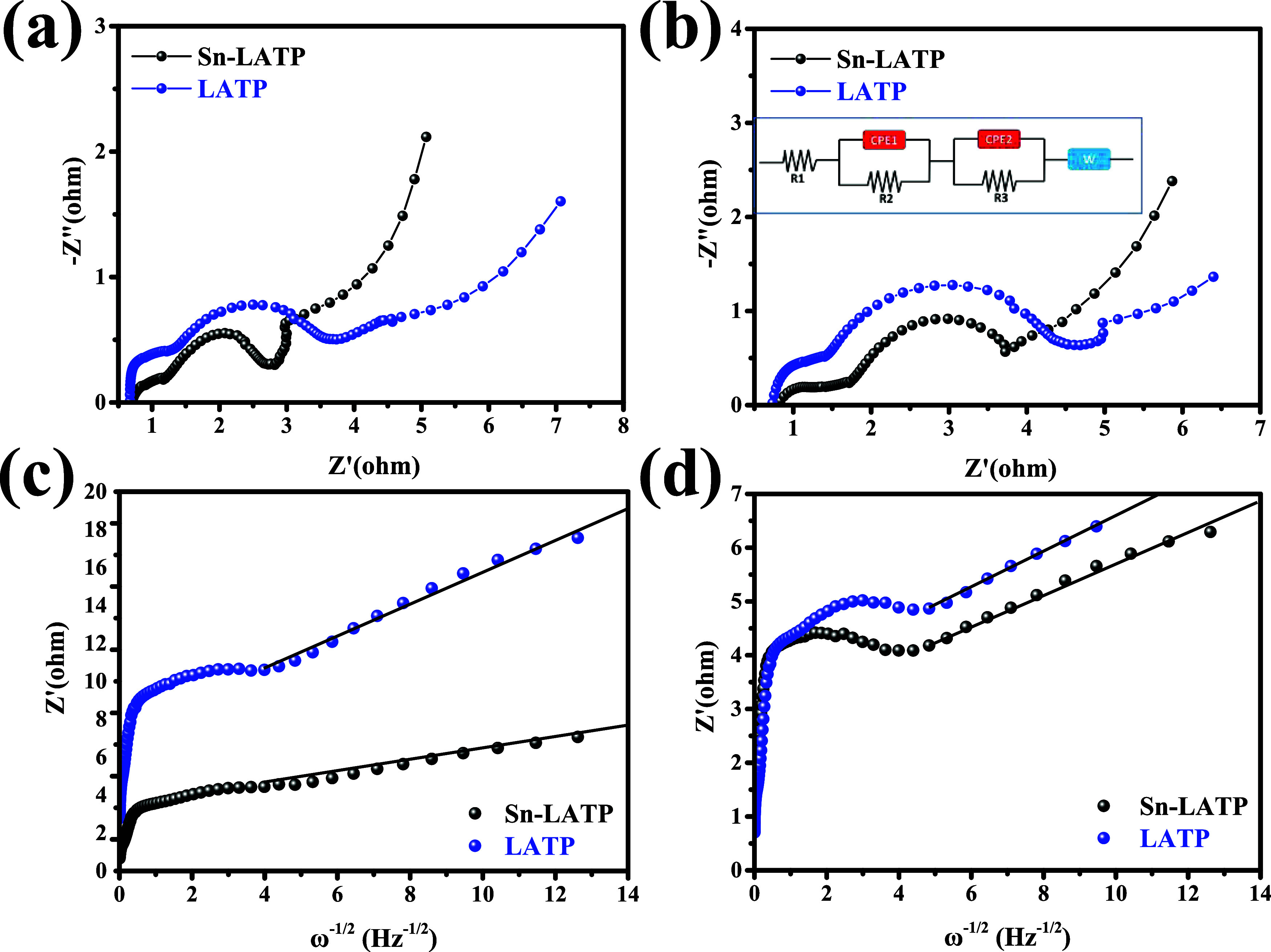
Nyquist plots of solid-state pouch cells at 3.6 V: (a) NCM523||Sn-LATP||MG11 and NCM523||LATP||MG11; (b) NCM622||Sn-LATP||MG11 and NCM622||LATP||MG11. Randles plots of pouch cells: (c) NCM523||Sn-LATP||MG11 and NCM523||LATP||MG11; (d) NCM622||Sn-LATP||MG11 and NCM622||LATP||MG11. The inset of (b) shows the proposed equivalent circuit.

Figure 7a,b illustrates that the Nyquist plots reveal an inclined line at approximately 45° to the Re *Z*’-axis, a consequence of the solid-state diffusion process of lithium ions within the electrodes.^48,49^ In the context of electrodes, Warburg impedance can be expressed as *W* = *k*_W_ ω^–1/2^ (1 – j), derived from the semi-infinite diffusion model.^42^ Here, *k*_W_ denotes the Warburg coefficient, and ω (= 2π*f*) signifies the angular frequency (*f*).^50^ Figure 7c,d presents the “Randles plot”, Re *Z*’ versus ω^–1/2^, for all solid-state pouch cells. The two sets of linear plots observed for each pouch cell demonstrate the diffusion of lithium ions within a heterogeneous electrode structure. The diffusion coefficients of Li ions (*D*_Li_), determined from *k*_w_ values, were calculated and are listed in Table 3. The *D*_Li_ values for the NCM523||Sn-LATP||MG11, NCM523||LATP||MG11, NCM622||Sn-LATP||MG11, and NCM622||LATP||MG11 pouch cells are determined to be 2.18 × 10^–8^ cm^2^ s^–1^, 1.05 × 10^–8^ cm^2^ s^–1^, 1.47 × 10^–8^ cm^2^ s^–1^, and 7.45 × 10^–9^ cm^2^ s^–1^, respectively. Both cells fabricated with Sn-LATP powders show higher *D*_Li_ values, about 1.97–2.08 times higher than those of pristine LATP powders. This finding reveals a significant reduction in the ionic diffusion resistance. The improved diffusion coefficients, due to the Sn-LATP powders in the CSEs, can be attributed to the Sn dopants, resulting in a smaller LATP powder grain size and more grain boundaries, leading to more ionic conduction pathways. Thus, Sn-LATP powders not only promote ionic conduction in CSEs but also facilitate subsequent Li-ion transport through the NCM lattice structure via NCM-supported CSEs, significantly enhancing the rate capability.^51,52^ In conclusion, the EIS behavior influenced the Sn-doped LATP solid-state pouch cell performance predominantly through improved bulk Li^+^ transport and reduced electrolyte–cathode interfacial resistance, with other effects such as dispersion state, surface reactivity, and alterations to the electrolyte’s chemical environment playing subsidiary roles. Further, post-cycling morphological characterizations are shown in Figures S2 and S3 for the NCM523 and NCM622 cathode sheets, respectively. Each figure shows no lithium dendrite growth in the Sn-LATP-containing CSEs, which can be attributed to the more homogeneous Li^+^ transport and reduced interfacial polarization generated by Sn doping. Compared with pristine LATP, Sn-LATP provides improved ionic conduction pathways, lower equivalent series resistance, and higher Li^+^ diffusion coefficients, which collectively decrease the local current-density accumulation at the electrolyte/electrode interface. This uniform Li^+^ flux is beneficial for minimizing uneven Li deposition during repeated cycling. In addition, Sn incorporation modifies the LATP lattice, promotes smaller grain domains, and increases grain boundary-assisted Li^+^ migration pathways, while the relatively high electronegativity of Sn contributes to the structural stability of the NASICON framework. Therefore, the improved cycling stability and high Coulombic efficiency of Sn-LATP-based pouch cells are attributed to the synergistic effects of enhanced Li^+^ transport, reduced charge-transfer resistance, and stabilized interfacial contact.

**Table 3:** Diffusion Coefficients of Solid-state Pouch Cells Equipped with Different CSEs

electrode type	composite solid-state electrolyte	voltage (V)	*D*_Li_ (cm^2^ s^–1^)
NCM523	LATP	3.6	1.051 × 10^–8^
NCM523	Sn-LATP	3.6	2.181 × 10^–8^
NCM622	LATP	3.6	7.445 × 10^–9^
NCM622	Sn-LATP	3.6	1.465 × 10^–8^

To further evaluate the significance of the impedance reduction achieved by Sn-LATP, the present EIS results were compared with representative NASICON-type solid electrolytes reported in the literature. NASICON-type LATP and LAGP electrolytes are widely regarded as promising oxide-based solid electrolytes because of their relatively high Li^+^ conductivity, air stability, and thermal robustness. However, their practical application is frequently limited by high grain boundary resistance, poor solid–solid contact with electrodes, and large electrode/electrolyte interfacial impedance. For example, pristine LATP- and LAGP-based systems often require surface coatings, gel/liquid-assisted interlayers, polymer matrices, or compositional doping to reduce their interfacial resistance and improve their electrochemical stability. Reported strategies such as polymer/LATP composite electrolytes, LATP/LAGP bilayer structures, coated LATP surfaces, and doped LATP ceramics can effectively decrease interfacial resistance; however, many of these studies are based on ceramic pellets, Li symmetric cells, or coin cells, where the electrode area, electrolyte thickness, stack pressure, and contact conditions differ substantially from practical pouch cell configurations.

In this context, the low ESR values obtained for the present pouch cells demonstrate the practical advantages of Sn-LATP-containing CSEs. The ESR decreases from 4.5 Ω for NCM523||LATP||MG11 to 3.1 Ω for NCM523||Sn-LATP||MG11 and from 5.4 Ω for NCM622||LATP||MG11 to 4.2 Ω for NCM622||Sn-LATP||MG11. This reduction confirms that Sn-LATP effectively suppresses the combined contributions of the CSE bulk resistance, interfacial SEI resistance, and charge-transfer resistance. In addition, the calculated Li-ion diffusion coefficients increase from 1.05 × 10^–8^ to 2.18 × 10^–8^ cm^2^ s^–1^ for the NCM523 system and from 7.45 × 10^–9^ to 1.47 × 10^–8^ cm^2^ s^–1^ for the NCM622 system when pristine LATP is replaced by Sn-LATP. These values indicate that Sn doping promotes faster Li^+^ transport by modifying the NASICON framework, reducing the diffusion resistance, and improving the CSE/electrode interfacial pathway. Therefore, compared with conventional NASICON-type electrolyte designs that often rely on additional interfacial layers or liquid-assisted wetting, the Sn-LATP-based CSE provides an effective impedance reduction strategy directly applicable to solid-state pouch cells (Table S1).

The specific energy density of solid-state pouch cells operating at high C rates is known to significantly decrease in high-power density regimes. This is undesirable for future-generation electric vehicles and mobile tools demanding high-rate cyclability with a smaller footprint.^52,53^ The real-time performance of the as-prepared pouch cells was then analyzed. The specific energy and power density of pouch cells utilizing NCM523 and NCM622 cathodes were analyzed using Ragone plots. The Ragone plots, shown in Figure 8, clearly demonstrate that all solid-state pouch cells maintain a good energy density. The energy densities of all pouch cells ranged from 220 to 246 Wh kg^–1^ at a power density of 20 W kg^–1^. For comparison with high-power performance, the energy density of the NCM523||Sn-LATP||MG11 pouch cell reaches as high as approximately 132 Wh kg^–1^ at a power density of 717 W kg^–1^. In contrast, the energy density of the NCM622||Sn-LATP||MG11 pouch cell reaches as high as approximately 172 Wh kg^–1^ at a power density of 778 W kg^–1^. Such exceptional performance makes the highly conductive Sn-LATP powder a promising ionic conductor for high-performance batteries.

**Figure 8:**
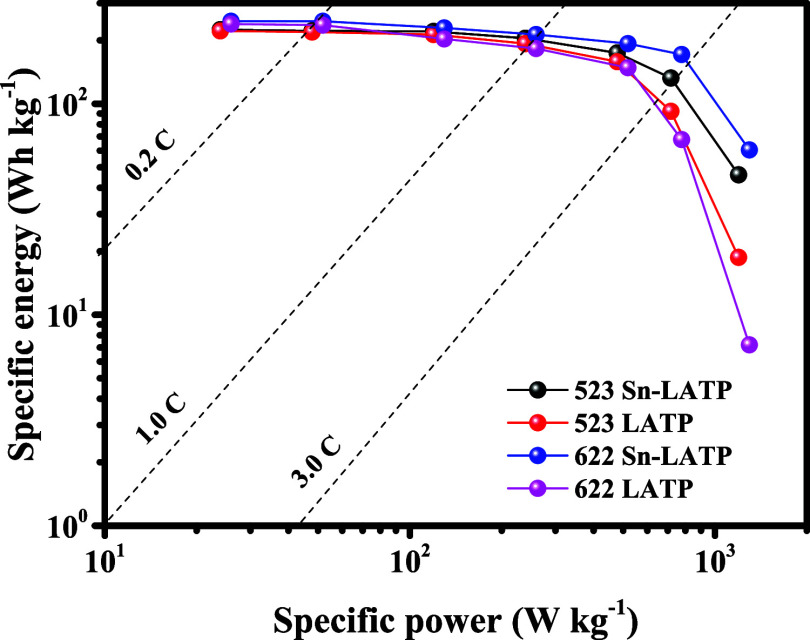
Ragone plots of different solid-state pouch cells equipped with NCM cathodes, artificial graphite anodes, and CSEs using pristine LATP and Sn-doped LATP powders.

## 4 Conclusions

This work has successfully demonstrated the manufacturing of assembled solid-state pouch cells using nickel-rich ternary cathode materials. Sn doping in LATP improved the Li-ion conductivity, rate performance, and cyclability of the pouch cells. The NCM622||Sn-LATP||MG11 pouch cell achieved a capacity retention of 91.1% with a high Coulombic efficiency (∼99.5%) over 200 cycles. The solid-state pouch cells delivered high energy densities ranging from 220 to 246 Wh kg^–1^. The energy densities of the NCM523||Sn-LATP||MG11 and NCM622||Sn-LATP||MG11 pouch cells were 132 and 172 Wh kg^–1^ at power densities of 717 and 778 W kg^–1^, respectively. Such exceptional performance highlights Sn-LATP powder as a promising ionic conductor for high-performance batteries. Although Sn-doped LATP demonstrated improved performance compared with pristine LATP, future studies should systematically optimize the Sn concentration to identify the most effective composition for enhancing the ionic conductivity, interfacial stability, and cycling performance, while extended long-term cycling under practical operating conditions will further validate the commercial reliability of Sn-LATP-based solid-state pouch cells beyond the present 200-cycle evaluation.

## Supplementary Material



## Notes

Synthesis of Sn-doped LATP powders (S1); assembly of solid-state pouch cells (S2); material and electrochemical characterization (S3); SEM images of (a) surface and (b) cross-section of the Sn-LATP slurry coated on the NCM622 cathode (Figure S1); top-view SEM images of NCM523 cathode sheets with (a, b) LATP and (c, d) Sn-LATP ceramics (Figure S2); top-view SEM images of NCM622 cathode sheets with LATP and Sn-LATP ceramics (Figure S3); comparison of NASICON-type solid-electrolyte strategies reported in the literature and present work (Table S1) (PDF)
