# Supplementary material for: Tin-Doped LATP Electrolytes Incorporated with Nickel-Rich Ternary Cathodes for Solid-State Pouch Cells Exhibiting High-Rate Capability and Excellent Cycling Stability
Source: ACS Omega. 2026 Jul 17;11(29):44326–35. doi: 10.1021/acsomega.6c04655 (PMC13425757; doi:10.1021/acsomega.6c04655)
Supplement: Supplementary file 1 [file ao6c04655_si_001.pdf]

Supporting information

Tin-doped LATP Electrolytes Incorporated with Nickel-rich Ternary Cathodes for Solid-State Pouch Cells with High-Rate Capability and Excellent Cycling Stability

Pradeep Kumar Panda[1,*], Yun-Ruei Huang[1], Siyong Gu[2], Pranjyan Dash[3], Congrui Jin[4], Jianlin Li[5,*], Chien-Te Hsieh[1, 6,*]

[1]Department of Chemical Engineering and Materials Science, Yuan Ze University, Taoyuan 32003, Taiwan

[2]Key Laboratory of Functional Materials and Applications of Fujian Province, School of Materials Science and Engineering, Xiamen University of Technology, Xiamen 361024, China

[3]Department of Chemical Engineering and Biotechnology, National Taipei University of Technology (Taipei Tech), Taipei 10608, Taiwan

[4]Department of Engineering Technology and Industrial Distribution, Texas A&M University, College Station, TX 77843, United States

[5]Applied Materials Division, Argonne National Laboratory, Lemont, IL 60439, United States

[6]Department of Mechanical, Aerospace, and Biomedical Engineering, University of Tennessee, Knoxville, TN 37996, United States

Corresponding author(s): Dr. P.K. Panda (rkpanda277@gmail.com), Prof. C.-T. Hsieh (E-mail: cthsieh@saturn.yzu.edu.tw and Dr. J. Li (jianlin.li@anl.gov).

S1. Synthesis of Sn-doped LATP powders

The synthesis of Sn-doped $Li_{1.3}Al_{0.3}Ti_{1.7}(PO_4)_3$ powders involved substituting Ti in the formula (y = 1.7) with Sn, reducing the Ti content to y= 1.4 and introducing Sn at 0.3. The sol-gel procedure is outlined as follows. All reagents were of analytical grade purity. Initially, a precursor containing concentric HNO_3 and $Ti(C_6H_9O)_4$, (Fluka, purity: 97 %) were well mixed in distilled water at a 1:4 volumetric ratio. Stoichiometric amount of $LiNO_3$ (Alfa, purity: 99%), $Al(NO_3)_3 \cdot 9H_2O$ (Alfa, purity: 98%), and $SnCl_2$ (Alfa Aesar, purity: 99%) were dissolved into a 0.2M aqueous solution of citric acid and stirred at 90 °C for 1 h to form a clear solution. Subsequently, citric acid ($C_6H_8O_7 \cdot H_2O$, Sigma, purity: 99.5 %) was added step-wise at ambient temperature, maintaining a molar ratio of citric acid to total metal ions at 4:1. A saturated solution of $NH_4H_2PO_4$ was then added to the homogeneous solution, and the pH was adjusted to 7 with $NH_3 \cdot H_2O$.

The solution was maintained at 90 °C for 5 h, forming a homogeneous emulsion, which was then dried at 120 °C to produce a dry gel, facilitating esterification between ethanol and citric acid. The obtained dry gel was heated to 180 °C for 4 h and then 250 °C for 5 h to promote pyrolysis. The Sn-doped LATP powders were further heated to 850 °C at a ramping rate of 5 °C min^{-1} in air and calcined at this temperature for 2 h. The powders were refined through ball-milling using a planetary mill (Fristch 77 Classic; Eritec Science Co., LTD) at 700 rpm for 90 min with 1-mm diameter Zr balls, maintaining a Zr ball to LATP powder ratio: 1:15. The refined Sn-doped LATP powders were dried at 120 °C for 2 h, resulting in the Sn-doped LATP sample (denoted as Sn-LATP). For reference, LATP powders without any dopants was designated as LATP.

S2. Assembly of solid-state pouch cells

The Assembly of solid-state pouch cells as follows. Ni-rich NCM523 and NCM622 cathodes (Shanghai Shanshan Technology Co., Ltd.) were prepared by mixing active material, poly(vinylidene fluoride) (PVDF) binder, and Super-P (Imerys Graphite & Carbon) in a weight ratio of 95.4:2.4:2.2 with NMP as the solvent. The slurry was uniformly coated on aluminum foil using a roll-to-roll coater, dried with warm air, and thickness-controlled to ~150 μm. The coated cathode sheets were vacuum-dried at 135 °C for 12 h to remove residual solvent, followed by compression (~200 $kg \cdot cm^{-2}$) to an average thickness of ~60 μm.

Artificial graphite anodes (MG11, China Steel Chemical Co.) were fabricated using a graphite: PVDF: Super-P weight ratio of 94.3:4.0:1.7 in NMP, following a process similar to cathode preparation but with copper foil as the current collector. Both cathode and anode sheets were cut to dimensions of ~46.5 × 31.5 mm^2. The CSE layer was laminated onto the cathode, and the composite was compressed and vacuum-baked at 140 °C for 24 h to remove residual NMP. The dried CSE coating layer has an average thickness of ~20 μm and about 3 wt.% LiTFSI salt was added into the composite cathode to facilitate Li^+-ion transport in SSLBs. The areal weight for cathode and anode was maintained at approximately 12.8 and 8.6 mg cm^{-2}, respectively. The nominal thickness of single-sided MG11 anode sheets was controlled within 70–80 μm. The anode-to-cathode capacity ratio was maintained at ~1.2. Finally, both NCM523||CSE||MG11 and NCM622||CSE||MG11 pouch cells were assembled in a glove box.

S3 Materials and electrochemical characterization

Microstructures of electrode materials were studied using scanning electron microscopy (SEM, JEOL JSM-7800F Prime) and high-resolution transmission electron microscopy (HR-TEM, JEOL 2100F). The crystalline structure of Sn-doped LATP samples was investigated using X-ray diffraction (XRD, Brucker D2 diffractometer with Cu target). Charge/discharge cycling studies of solid-state pouch cells were undertaken at varying C rates (0.1 to 5 C) within a voltage range of 2.8–4.3 V at ambient temperature. The polarization distribution in pouch cells built with different CSEs was also measured using electrochemical impedance spectroscopy (EIS, CH Instruments 608C). EIS measurements were carried out at different potentials within a frequency range from 100 kHz to 1 mHz. An argon-filled glove box (Braun Co. Ltd.) was employed for pouch cell construction.

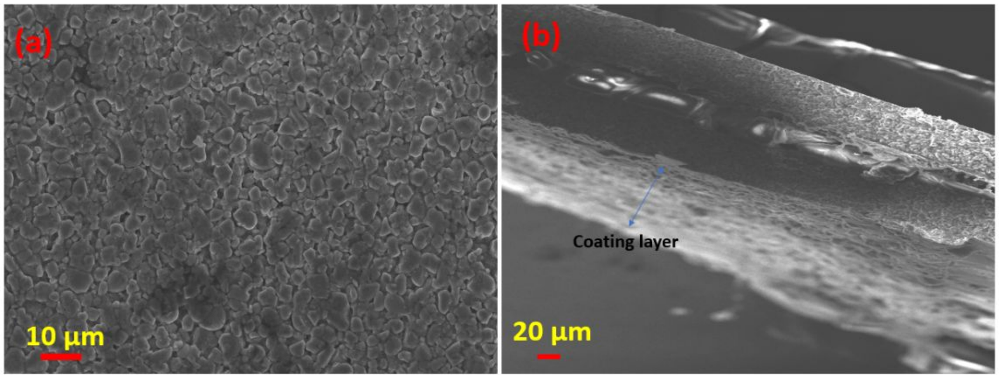

Figure S1: SEM image of (a) surface and (b) cross-section of the Sn-LATP slurry coated on the NCM 622 cathode.

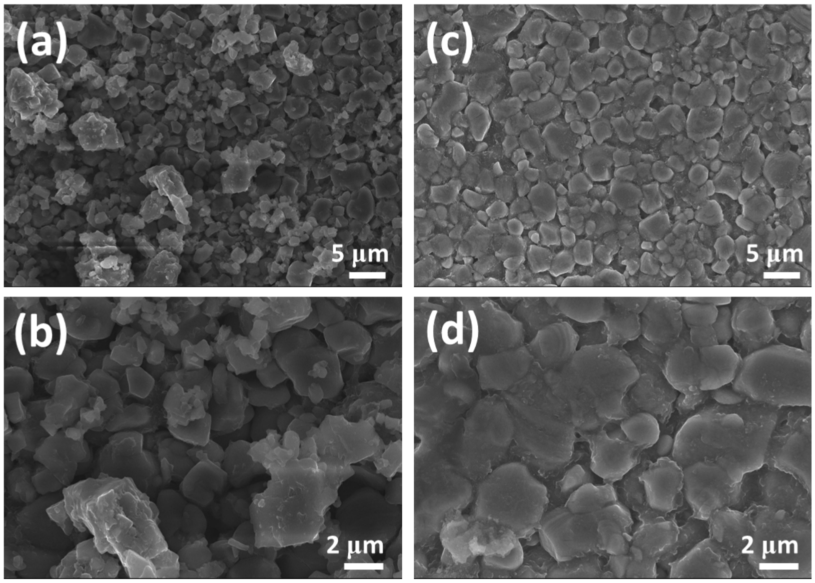

Figure S2. Top-view SEM images of NCM523 cathode sheets with (a,b) LATP and (c,d) Sn-LATP ceramics. The cathode sheets were placed from solid-state pouch cells after cycling at ambient temperature.

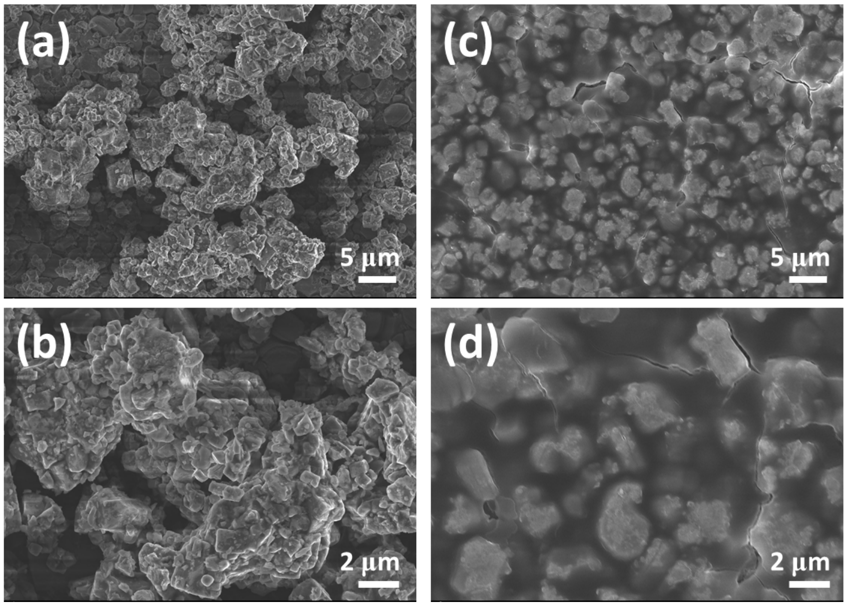

Figure S3. Top-view SEM images of NCM622 cathode sheets with (a,b) LATP and (c,d) Sn-LATP ceramics. The cathode sheets were placed from solid-state pouch cells after cycling at ambient temperature.

Table S1: Comparison of NASICON-type solid electrolyte strategies reported in the literature and the present work.

**Electrolyte system**	**Cell/configuration**	**Main impedance-related observation**	**Key limitation or feature**
Pristine LATP	Ceramic pellet or solid-state cell	High grain-boundary and interfacial resistance are commonly observed	Poor solid–solid interfacial contact and possible instability toward Li metal
LAGP-based NASICON electrolyte	Ceramic or protected-interface cell	High ionic conductivity can be achieved, but interfacial resistance remains a major concern	Often requires coating or interfacial modification
LATP/polymer composite electrolyte	Coin cell or Li symmetric cell	Polymer matrix improves contact and reduces interfacial impedance	Conductivity and resistance depend strongly on polymer type, filler loading, and temperature
Coated LATP or LATP with gel/liquid interlayer	Li symmetric or full cell	Interfacial resistance can be significantly reduced by improving wetting/contact	Additional interlayer may complicate cell design and reduce true solid-state character
Sn-doped LATP ceramic/CSE	Ceramic or composite electrolyte cell	Sn doping enhances ionic transport by modifying Li^+ migration pathways	Prior studies are mainly focused on electrolyte-level or coin-cell evaluation
Present Sn-LATP CSE	NCM523/NCM622 solid-state pouch cells	ESR decreases from 4.5 to 3.1 Ω for NCM523 and from 5.4 to 4.2 Ω for NCM622; D_{Li} increases by approximately twofold	Demonstrates impedance reduction in practical pouch-cell configuration
